# Community-Based Mental Health Promotion and Public Policy Integration: A Scoping Review (1990–2024)

**DOI:** 10.3390/healthcare14131931

**Published:** 2026-07-01

**Authors:** Alexandra Judith Caycedo Sabaraín, Favio Cala Vitery, Laura Inés Plata Casas

**Affiliations:** 1 PhD Public Policy and Management Modeling, Faculty of Natural Sciences and Engineering, Universidad de Bogotá Jorge Tadeo Lozano, Bogotá 110311, Colombia; favio.cala@utadeo.edu.co; 2 National Superintendence of Health, Carrera 68A No. 24B-10, Tower 3, Floors 4, 9 and 10, Plaza Claro Building, Bogotá 110931, Colombia; lplatac71@gmail.com

**Keywords:** mental health promotion, community health, scoping review, public health policy, health systems, community health services, mental health

## Abstract

**Highlights:**

**What are the main findings?**

- Community-based mental health promotion strategies are diverse and primarily implemented in school and community settings, with a focus on psychosocial skills, resilience, and social support.
- Integration with public health systems and policy frameworks is uneven, with limited documentation on sustainability and long-term outcomes.

**What are the implications of the main findings?**

- Strengthening the articulation of community-based interventions within healthcare systems may improve equity, sustainability, and population impact.
- The findings provide actionable evidence to inform mental health policy development, particularly in low- and middle-income countries.

**Abstract:**

**Background:** Community-based mental health promotion has gained increasing relevance as a strategy to strengthen population well-being and complement formal healthcare services. However, existing initiatives remain fragmented, and their integration into health systems and public policy frameworks has not been systematically examined. This scoping review aimed to map community-based mental health promotion strategies and analyze their alignment with public health systems and policy frameworks. **Methods:** A scoping review was conducted following the Joanna Briggs Institute methodology and reported according to the PRISMA-ScR guidelines. Searches were conducted in April 2025 across major databases, including Scopus and PubMed, covering studies published between 1990 and 2024. The retrieved records were subsequently reviewed and analyzed by the researchers between 1 May 2025 and September 2025 Documents published after 2024 were used only as contextual or policy references and were not included in the review corpus. Eligibility criteria were defined using the Population–Concept–Context framework. Two reviewers independently screened records and extracted data. **Results:** A total of 3799 records were identified, of which 76 studies met the inclusion criteria. Most interventions were implemented in school (18.4%) and community (21.1%) settings and focused on strengthening psychosocial skills, social support, and resilience. Common intervention components included community participation, cultural adaptation, and facilitator training. Several strategies were linked to broader public health frameworks, such as primary health care, intersectoral action, and social determinants of health. Reported outcomes were generally positive, although evaluation methods and indicators varied widely. **Conclusions:** Community-based mental health promotion interventions represent a valuable complement to healthcare systems, particularly in resource-constrained settings. Strengthening their integration into public policies and health system planning may improve sustainability, equity, and population impact. This review highlights key gaps in implementation and evaluation and provides evidence to inform decision-making in community health, prevention, and mental health policy development.

## 1. Introduction

Mental disorders and mental health problems constitute a growing challenge for public health systems worldwide. The COVID-19 pandemic intensified this impact, increasing the burden of disease, deteriorating psychosocial well-being, and reducing healthy life years [1,2,3].

According to the World Health Organization (WHO), more than one billion people worldwide currently live with a mental health condition, with anxiety and depression being the most prevalent disorders [4]. The pandemic led to a 25% increase in depressive and anxiety disorders in just one year [5].

The impact has been particularly severe among young people, with suicide becoming the third leading cause of death among individuals aged 15 to 29 years. In 2021, approximately 727,000 people died by suicide, and many more attempted it. Adolescence represents a critical stage, as one in seven adolescents experiences a mental disorder, and half of all mental disorders in adults begin before the age of 18 [6,7,8,9,10].

In Latin America, only one in five people with mental disorders receives treatment, reflecting a critical gap in access to services. In addition, stigma, discrimination, and insufficient investment exacerbate the situation, leading to consequences such as unemployment, poverty, social exclusion, and family burden [4,11].

The WHO and the Pan American Health Organization (PAHO) have warned that mental health problems not only affect individual quality of life but also reduce economic productivity, increase health expenditures, and hinder social development [4,11,12].

To address the high prevalence and profound social impact of mental disorders, it is necessary to rethink traditional interventions centered on the clinical model, promoting broader, more effective, and community-oriented approaches. Evidence shows that most countries concentrate their resources on specialized and hospital-based care, while mental health promotion and community prevention strategies receive significantly less investment [13,14].

It is necessary to define and differentiate three fundamental concepts in mental health—promotion, prevention, and treatment—given that the present review focuses exclusively on the promotion approach. Mental health promotion, according to the World Health Organization (WHO), is defined as “the process of enabling individuals and communities to increase control over the determinants of mental health and improve their mental well-being” [15]. From this perspective, promotion adopts a broad, population-based approach that seeks to strengthen protective factors such as resilience, life skills, and social support, aiming not only to prevent illness but also to create conditions that allow individuals and communities to thrive.

In contrast prevention, from a public health perspective, is defined by UNICEF as “interventions aimed at reducing the incidence, prevalence, and recurrence of mental disorders, as well as decreasing risk factors and strengthening protective factors before the problem manifests” [16]. Prevention operates across primary, secondary, and tertiary levels and focuses specifically on reducing risk and mitigating the onset of mental health conditions.

Finally, mental health treatment comprises “the set of clinical, psychosocial, and community interventions directed at individuals with mental disorders or significant psychological distress, with the aim of reducing symptoms, improving functioning, and promoting recovery” [17]. This distinction clarifies the difference between individual-level clinical care and population-level strategies.

This distinction is essential for the formulation of coherent public policies, as it guides the allocation of resources toward population-based promotion strategies rather than focusing solely on risk reduction.

Although these approaches share the goal of improving mental health, they differ in scope and orientation yet remain interdependent. Within this framework, community-based mental health care is understood as an approach that integrates these components in participatory, context-sensitive settings. In this review, community-based interventions were considered those involving active community participation in their design, implementation, or delivery, ensuring alignment between conceptual definitions and inclusion criteria. The WHO and PAHO have emphasized that health systems must advance toward people-centered, human-rights-based models that integrate mental health into primary health care and community settings. These models help reduce stigma, strengthen local support networks, and empower communities as active agents in mental health care [13,17,18].

Furthermore, community-based interventions—such as integrative community therapies, listening circles, and mental health education programs—have been shown to improve emotional well-being, reduce social isolation, and prevent severe outcomes such as suicide, particularly among vulnerable populations [14,18].

Persisting with an exclusively clinical approach focused on specialized and hospital-based care limits preventive impact and generates higher social and economic costs. There is a need to redistribute resources toward promotion, prevention, and community participation as pillars of a sustainable and equitable public policy [13,14].

A review of the available literature did not identify previous reviews that comprehensively integrate community-based mental health promotion strategies with public policy frameworks. Existing evidence primarily focuses on isolated interventions without analyzing their articulation with such frameworks, highlighting a knowledge gap that justifies the conduct of a scoping review.

In Colombia, the National Mental Health Policy [19] and the Ten-Year Public Health Plan recognize these gaps; however, there remains limited evidence regarding the effectiveness of community-based strategies in reducing suicidal behavior.

In this context, the objective of this scoping review was to analyze national and international theoretical models and approaches to mental health promotion, identifying how public policy is incorporated into community-based interventions. To this end, studies evaluating interventions designed to promote mental health in communities were examined. Based on the PCC framework (Population, Concept, and Context), the review focused on community populations (P), analyzed community-based mental health promotion interventions as the concept of interest (C), and considered their development within public policy and health system contexts across different countries (C).

For the purposes of this study, promotional strategies are understood as interventions aimed at strengthening protective factors, psychosocial skills, emotional well-being, and community participation, excluding clinical treatment or rehabilitation actions.

## 2. Materials and Methods

The scoping review process was conducted in accordance with the JBI Manual for Evidence Synthesis (Joanna Briggs Institute), internationally recognized as a methodological reference for scoping reviews. This approach enables the rigorous integration and appraisal of different types of evidence—quantitative, qualitative, and mixed methods—ensuring the validity and comprehensiveness of the analysis. The application of this methodology supports a holistic perspective that goes beyond the traditional clinical domain and allows for the incorporation of community, social, and policy dimensions in mental health promotion [20]. In addition, the review was reported following the PRISMA-ScR checklist recommendations [21].

The review protocol was registered in the Open Science Framework (OSF) under the DOI identifier 10.17605/OSF.IO/YA35G.

The PCC (Population–Concept–Context) framework was used to formulate the research question and define the eligibility criteria [22]. The period considered ranged from 1990 to 2024 [23]. Documents published in 2025 were not included in the analytical sample but were used exclusively as contextual inputs to support the interpretation of policy frameworks and recent developments. The framework was applied according to the following components:

P (Population): Individuals from the general community or specific subgroups, such as adolescents, older adults, or people in situations of social vulnerability.

C (Concept): Mental health promotion interventions and community-based strategies aimed at strengthening psychosocial well-being. Outcomes related to overall well-being, increased mental health knowledge, changes in healthy behaviors, and reduction of risk factors were considered [24].

To ensure conceptual and methodological consistency in this study, mental health constructs (including anxiety, depression, suicidal behavior, resilience, psychosocial functioning, and well-being) are approached from a public health and mental health promotion perspective rather than from a diagnostic framework based on DSM-5 criteria. Consistent with the objective of the scoping review, which focuses on community-based interventions aimed at strengthening well-being and protective factors, the included studies primarily rely on dimensional indicators, such as symptom levels, subjective well-being, and psychosocial capacities. Within this framework, anxiety and depression are considered expressions of emotional distress measured through validated scales, without necessarily implying a clinical diagnosis, while suicidal behavior is addressed from a public health perspective that encompasses ideation, attempts, and associated risk factors. This approach aligns with contemporary models that conceptualize mental health as a continuum rather than as a set of categorical diagnoses. This operationalization guided both study selection and data extraction. C (Context): Interventions developed in community settings within the framework of public policies and health systems across national and international contexts. Conventional mental health promotion interventions—such as general informational campaigns or individual psychological care without active community participation—were considered as contextual comparators.

### 2.1. Inclusion Criteria

Empirical studies with quantitative, qualitative, and mixed-methods designs—including experimental, quasi-experimental, and observational studies—as well as systematic or integrative reviews, program evaluations, and evaluations of community-based interventions were included. Documents related to policies, strategies, or mental health promotion programs were also considered. The focus of interest was strictly community-based, understood as interventions implemented in social and territorial contexts aimed at promoting mental health. Studies grounded in theoretical models or approaches applied to community mental health, published in indexed journals between 1990 and 2024, in Spanish, English, or Portuguese, were selected.

For the purposes of this review, “community-based” refers to interventions implemented outside clinical settings that also involve active community participation in their design, implementation, or delivery. This includes participatory approaches, co-creation processes, and interventions led by or embedded within community networks. Interventions that were non-clinical but lacked meaningful community engagement were not considered eligible.

### 2.2. Exclusion Criteria

Studies that did not address strategies directly related to mental health were excluded, as were those focused exclusively on the promotion of healthy habits without an explicit link to this topic. Research aimed solely at general well-being without a defined theoretical framework or outcome evaluation was also excluded, as were studies centered on clinical, psychiatric, or individual treatment without a community component. In addition, studies conducted in workplace, school, or institutional settings without community articulation were excluded, along with narrative reviews lacking explicit methodology, opinion papers, editorials, letters to the editor, and abstracts without empirical data.

Grey literature not published in indexed journals—such as theses, dissertations, technical reports, conference proceedings, preprints, manuals, and institutional documents—, was excluded. However, normative and public policy documents were included as contextual inputs for the analysis of regulatory frameworks.

### 2.3. Geographic Scope

Studies conducted in national, regional (Latin America), and international contexts were considered, with particular attention to countries with consolidated community mental health policies.

### 2.4. Search Strategy and Reference Management

References were searched for studies published between 1 January 1990 and 31 December 2024. Subsequently, the review process by the researchers was conducted between 1 May and 25 September 2025. Any sources published after this period were considered only for contextual or background purposes and were not included in the systematic selection process.

Controlled MeSH and DeCS descriptors were used, combined through Boolean operators (AND, OR). The terms were organized into eight thematic groups aligned with the study’s explanatory variables (mental health promotion, public policies, theoretical models, community intervention, social determinants, national and international approaches, vulnerable communities, and public policy strategies). Search equations were adapted to each database.

Reference management was conducted using Mendeley Desktop 1.19.8 software, which enabled duplicate removal, thematic organization, and record traceability. The study selection process followed the PRISMA-ScR guidelines. A total of 3799 records were identified through database and supplementary searches, including Scopus (*n* = 684), Cochrane (*n* = 43), BVS/VHL (*n* = 2966), PubMed (*n* = 10), SciELO (*n* = 32), and other sources (*n* = 64). After the removal of duplicates, incomplete records, and studies without full-text access, 3685 records remained for screening. After title and abstract screening, 3505 records were excluded.

After title and abstract screening, 180 records were considered potentially eligible and were sought for full-text retrieval. Of these, 104 records could not be retrieved. The remaining 76 full-text articles were assessed and all met the predefined inclusion and quality criteria, resulting in a final sample of 76 studies included in the scoping review.

**Figure 1:**
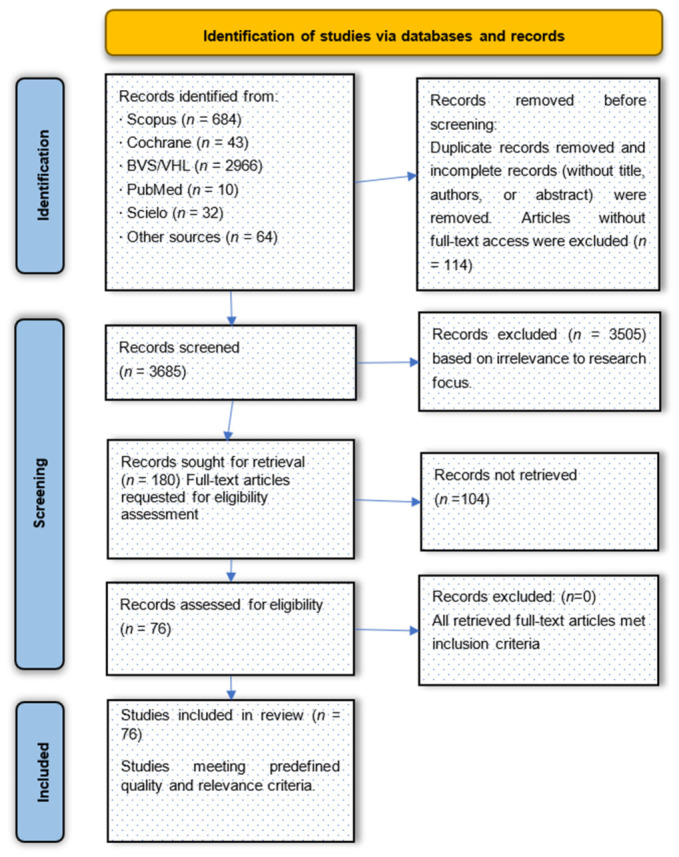
PRISMA-ScR flow diagram of study selection. Source: Authors’ own elaboration based on the PRISMA flow diagram derived from the scoping review process.

The study selection process is summarized in the PRISMA-ScR flow diagram presented in Figure 1 [21]. This scoping review is reported in accordance with the PRISMA-ScR guidelines, and the completed PRISMA-ScR checklist is provided as Supplementary Material (File S1: PRISMA-ScR Checklist).

The full search strategies for each database, including Boolean strings, filters, and limits, are provided in Supplementary Material File S2.

### 2.5. Quality Appraisal

Methodological quality was assessed using the Joanna Briggs Institute (JBI) critical appraisal tools, selecting the appropriate checklist according to each study design. Two reviewers independently evaluated all studies, and disagreements were resolved through consensus to ensure reliability.

The appraisal considered key aspects such as coherence between objectives and study design, clarity in the description of interventions, appropriateness of data collection and analysis methods, and consistency of reported results. Based on these criteria, studies were classified as high, upper-medium, medium, or low quality.

This classification provided a flexible yet systematic framework to assess heterogeneous evidence (quantitative, qualitative, and mixed methods), facilitating comparability while maintaining sensitivity to contextual relevance. It also ensured a transparent and reproducible evaluation process consistent with JBI guidance for scoping reviews.

### 2.6. Scoping Review Registration

The protocol for this scoping review was prospectively registered in the Open Science Framework (OSF) on 18 April 2026. The registration record is available at https://osf.io/ya35g under the DOI identifier 10.17605/OSF.IO/YA35G.

### 2.7. Specific Scoping Review Methodology

Once the search and importation of records into the Mendeley reference manager were completed, a systematic data-cleaning process was conducted to ensure information quality and relevance. Duplicate records were removed using Mendeley’s automatic detection function, complemented by manual verification of matches in title, authorship, year, and DOI.

Record completeness was then assessed, and incomplete entries (lacking title, authors, or abstract) and those without access to full text were excluded. Initial exclusion criteria were applied, removing articles that did not meet basic requirements: languages other than Spanish, English, or Portuguese; hospital-based clinical studies; and documents not published in indexed journals.

The refined records were subsequently organized into thematic folders according to explanatory variables (mental health promotion, public policies, community intervention, theoretical models, and social determinants). This process reduced the initial total of 3799 records to a refined dataset for the title and abstract screening phase.

Study selection was conducted in two stages. In the first stage, all articles were distributed among the researchers for title and abstract screening, applying exclusion and refinement criteria. In the second stage, studies deemed relevant underwent full-text review to confirm final inclusion in the scoping review.

Integration with public policy was operationalized as any explicit linkage to national or regional mental health plans, intersectoral governance mechanisms, financing or workforce provisions, and monitoring and evaluation frameworks described in the included sources.

### 2.8. Data Extraction and Categorization

A data extraction matrix was designed to systematize information on intervention type, population, country, methodology, and main findings. Methodologies were classified into predefined categories: quantitative pre–post, quantitative and systematic reviews, quasi-experimental with control group, controlled trial, applied qualitative study, study protocol, experimental design, scoping review, systematic review, and others.

Populations were categorized to facilitate comparative analysis into the following groups: children and adolescents, adults, vulnerable populations, community members, health professionals, and others.

### 2.9. Narrative Synthesis and Thematic Analysis

Results were organized according to promotional strategies, identifying gaps, barriers, and facilitating factors. Tables and figures were developed to display the distribution of methodologies and populations, enabling a comparative overview of applied approaches.

Due to the diversity of study designs, populations, contexts, and interventions, a narrative and thematic approach was used. Studies were grouped by key features to identify common patterns and implementation factors, avoiding direct comparisons. The focus was on conceptual coherence, consistent with the exploratory nature of scoping reviews.

Methodological quality assessment was conducted, as previously noted, following JBI guidelines. For this purpose, an extraction matrix including the corresponding variables was constructed, ensuring traceability and transparency of the analytical process. Quality was categorized as High, Upper-Medium, Medium, or Low. The complete matrix is presented in Supplementary Material File S1, following the JBI extraction structure: author, year, country, design, population, type of intervention, results, quality, and alignment with objectives.

### 2.10. Article Review Process

The operational review began on May 1 and concluded in September 2025 through a systematic process of title and abstract reading, aimed at identifying study relevance based on the research question structured under the PCC framework (Population–Concept–Context), as recommended by JBI for scoping reviews.

A previously described extraction matrix, designed in an Excel spreadsheet, was used to record the document link, title, abstract, and preliminary inclusion decision. Each article was labeled as “Accepted” or “Excluded” according to its relevance and methodological adequacy.

Retrieved documents were organized in Mendeley, classified by author, and distributed among researchers for detailed analysis. As the review progressed, consensus-based decisions were made regarding inclusion or exclusion, and articles were archived in labeled folders, ensuring traceability and organization for subsequent phases.

A total of 3685 documents were reviewed, initially through title and abstract screening and, in some cases, full-text reading, guided by a set of orienting questions aligned with the first specific study objective, including:

What mental health promotional strategies have been implemented at the community level?

Which community strategies have proven effective in promoting mental health in urban populations?

What indicators are used to evaluate the effectiveness of these strategies?

What variables or characteristics influence implementation?

What theoretical models or promotional approaches are applied to mental health in different national and international contexts?

What contextual factors (cultural, economic, political) influence the effectiveness of community interventions?

How have community-based mental health promotion strategies been incorporated into public policies?

What regulatory frameworks or national/international plans support these interventions?

What role do community actors (leaders, organizations, local networks) play in the implementation of mental health public policies?

At the end of this first phase, articles presenting evidence of the effectiveness of community-based mental health promotion strategies were filtered in. Effectiveness was evidenced by changes in mental well-being, strengthening of protective factors, and psychosocial functioning, measured using validated instruments such as mental well-being, resilience, self-efficacy, and social support scales, complemented by implementation and community context indicators. In this context, effectiveness refers to the degree to which community mental health promotion strategies achieve observable improvements in mental well-being, strengthen protective factors and individual and collective capacities, and reduce psychosocial risks when implemented under real-world conditions across diverse community settings.

### 2.11. Ethical Considerations

As this study is a scoping review based exclusively on previously published literature, it did not involve the collection of primary data from human participants. Therefore, ethics approval and informed consent were not required.

The review was conducted in accordance with the principles of research integrity, transparency, and proper citation of sources. Normative and policy documents were used solely for contextual and analytical purposes.

## 3. Results

A total of 3799 records were identified. Following the screening and eligibility assessment process, 76 studies were included in the final synthesis (Figure 1. PRISMA-ScR flow diagram of the study selection process). The general characteristics of the included studies are presented in Table 1.

Although the results reveal consistent trends in the implementation and effects of community-based strategies, they should be interpreted in light of the methodological diversity of the included studies. The mix of quantitative, qualitative, mixed-methods studies, and reviews limits direct comparability. Accordingly, results are presented as trends, patterns, and intervention typologies rather than comparable effectiveness estimates, with a focus on common approaches, implementation mechanisms, and their links to health systems and policy frameworks.

Table 2 presents an integrated analytical summary. This table enhances the analytical synthesis by facilitating comparison across studies in terms of intervention typologies, implementation mechanisms, and reported outcomes, complementing the detailed data presented in Supplementary Material File S1.

1. General characterization of included studies

The included studies met the PCC framework criteria and documented community-based mental health promotion strategies implemented across diverse contexts and populations. Based on the extracted information, interventions were classified according to their strategic typology, enabling a comparative analysis of the identified approaches. The thematic distribution of strategies is presented in Table 3.

2. Thematic classification of promotional strategies

**Table 3:** Typology of community-based mental health promotion strategies (*n* = 76).

Type of Strategy	*n*	%
School-based	14	18.4
Community-based	16	21.1
Digital and technological	3	3.9
Physical activity and exercise	7	9.2
Psychological and emotional	16	21.1
Cultural diversity	2	2.6
Other (workplace and complementary)	18	23.7
Total	76	100.0

Source: Authors’ own elaboration based on the scoping review conducted. Note: Percentages were calculated based on the total number of included studies (*n* = 76) and are presented with one decimal place to avoid rounding inconsistencies.

3. Synthetic description by strategic typology

School-based strategies (18.4%): Interventions implemented in schools and universities aimed at strengthening socio-emotional skills, resilience, and mental health literacy [24,25,26,27,28].

Community-based strategies (21.1%): Participatory actions developed in collective spaces, focused on strengthening social support networks and community resilience [29,30,31,32,33].

Digital and technological strategies (3.9%): Use of virtual platforms, applications, and online resources designed to expand access to mental health promotion interventions [28,34].

Exercise and physical activity (9.2%): Initiatives aimed at promoting mental well-being through physical activity, recreation, and the use of public spaces as settings for community intervention [35,36,37].

Psychological and emotional strategies (21.1%): Interventions based on mindfulness approaches, positive psychology, and psychosocial support groups, aimed at strengthening coping skills and emotional regulation [38,39].

Cultural diversity strategies (2.6%): Mental health promotion programs culturally adapted to specific contexts, integrating local knowledge and intercultural approaches [40,41].

Other strategies (23.7%): Interventions developed in workplace settings and complementary approaches that do not fall within the previous categories but contribute to community mental health promotion [42,43].

4. Implementation variables and intersectoral articulation

Across the analyzed contexts, the successful implementation of community-based interventions consistently depended on several key factors, including the training of community facilitators, cultural adaptation, intersectoral coordination, and the operational sustainability of strategies. Studies such as the iCARE-R model [44], teacher training initiatives in Uganda [45] and task-shifting experiences in vulnerable communities [42] illustrate how these factors are integrated across diverse settings to strengthen community mental health.

5. Linkage with public policy and social determinants

Regarding the articulation between community strategies and public policy, several theoretical and normative frameworks guiding the integration of these interventions into broader health systems and social policies were identified. The analysis also examined whether the included interventions aligned with operational frameworks for scaling up, such as the WHO mhGAP Community Toolkit, particularly in low-resource and high-inequality settings.

The review identified multiple frameworks linking community mental health strategies with inclusive public policy development. Notably, the proportionate universalism approach proposed by some authors promotes graduated interventions according to population needs [46]. Intersectoral models and the social determinants of health approach were also recognized as key foundations for cross-sectoral action, as outlined by several other authors [47,48,49]. Furthermore, recommendations to integrate the community mhGAP into national mental health plans, highlights the relevance of this framework as an operational guide in local contexts [50,51].

6. Distribution by target population

The identified community-based strategies targeted six specific population groups, with a proportional distribution reflecting the diversity of mental health approaches. Children and adolescents accounted for 37% of interventions, followed by adults at 26%. Vulnerable populations—including people affected by displacement, extreme poverty, or disabilities—represented 17% of strategies. Fourteen percent targeted communities in general, while 3% focused on health professionals and another 3% on other unspecified groups. This segmentation highlights the breadth of the community approach and its adaptability to diverse population contexts.

7. Integrated synthesis of findings

Overall, the analyzed community-based strategies demonstrated positive effects across multiple dimensions of mental health, including improvements in self-efficacy, resilience, and coping skills. A reduction in social isolation and a strengthening of individual and collective protective factors were also reported. The reviewed evidence highlights the feasibility of the community mhGAP as an effective operational framework for implementing interventions in local settings, reinforcing its potential as a key tool for community-based mental health promotion.

## 4. Discussion

From a scoping review perspective, these findings provide a comprehensive mapping of community-based mental health strategies, allowing for the identification of key patterns and implications for policy and practice. When grounded in robust theoretical models and articulated with inclusive public policies, these strategies generate significant impacts on emotional well-being, particularly among populations in vulnerable situations. The reviewed interventions show consistent improvements in self-efficacy, resilience, coping skills, and reductions in social isolation, especially in school and community settings.

The largest proportion of interventions was concentrated in community (21%) and school (19%) environments, reflecting a global emphasis on strengthening socio-emotional skills and support networks within collective spaces. In contrast, a low representation of digital strategies (4%) and culturally oriented interventions (2%) was observed, despite technological changes and increasing multiculturalism. This represents a relevant gap in the available evidence.

The community component of the Mental Health Gap Action Programme (mhGAP) emerges as a promising framework for expanding mental health responses beyond primary care. However, as noted by some authors [52,53] evidence on its community-level implementation remains limited, particularly regarding sustainability, territorial reach, and effective outcomes.

### 4.1. Implications for Public Policy

Regarding public policy implications, the findings support the need to formally integrate community-based mental health promotion into national health plans, under intersectoral, participatory, and human rights-based approaches. Several studies propose relevant frameworks for this integration, such as the proportionate universalism approach [46] and the focus on social, cultural, and structural determinants of mental health [47,48].

Similarly, agree that mental health promotion should constitute a structural component of national plans, with sustained funding, clear governance, and community participation, aligned with the guidelines of the community mhGAP, which promotes service expansion through local economic and human resources [50,51].

### 4.2. Critical Implementation Factors

The review identifies critical variables for the effective implementation of community interventions. These include facilitator training, intersectoral articulation, contextual adaptation, and operational sustainability. McAllister et al. [44] demonstrate that training in solution-focused communication and coordinated work between the education and health sectors were decisive for the success of the iCARE-R program.

Some authors reinforce this evidence by showing that task shifting to teachers and community actors allows for expanded coverage without compromising quality [54]. Meanwhile, document common structural barriers in low- and middle-income countries, such as institutional fragmentation, resource scarcity, and the absence of clear regulatory frameworks [50]. These limitations align with the observations previous observations emphasizing the need to assess dimensions such as acceptability, adoptability, fidelity, and sustainability in the implementation of the community mhGAP [52].

### 4.3. Integration with Health Systems and Policy Frameworks

Building on these findings, an integrative perspective is proposed to better understand how these interventions are incorporated into health systems.

Beyond the description of identified strategies, the findings indicate that the integration of community-based mental health interventions into health systems and policy frameworks is heterogeneous and occurs at different levels. Three main forms of integration were identified: (i) informational integration, where interventions are conceptually aligned with health promotion frameworks but lack operational linkage to health systems; (ii) operational integration, where strategies are implemented in coordination with existing services, particularly within primary care or community programs; and (iii) structural integration, where interventions are formally embedded within public policies, national plans, or care models, including mechanisms for financing, governance, and evaluation.

Important differences were observed across contexts. In high-income countries, integration tends to be more structural, supported by established institutional frameworks and policy-driven programs. In contrast, in low- and middle-income countries, community-based interventions often function as complementary or substitute strategies in response to gaps in formal systems, relying on approaches such as task shifting, intersectoral action, and frameworks like the community mhGAP. In these settings, integration is typically more flexible and less formally institutionalized.

Across studies, effective integration was associated with several recurring elements, including intersectoral collaboration, training of community facilitators, cultural adaptation, and the presence of scalable programmatic frameworks. However, despite positive outcomes in well-being, resilience, and social support, a persistent gap remains between implementation and full system integration. This is reflected in limited evidence on financing, monitoring, and standardized evaluation.

Overall, the findings suggest that the impact of community-based mental health strategies depends not only on their design, but also on their degree of integration into health systems and policy structures. Advancing toward sustainable and scalable models requires strengthening governance, financing, and intersectoral coordination mechanisms.

These findings reflect the inherent complexity of community-based mental health interventions across diverse contexts. This variation captures different levels of evidence—from practical implementation to synthesized findings—particularly in fields with limited experimental data. However, results should be interpreted as patterns and trends rather than direct comparisons of effectiveness. In this sense, the value of this study lies in integrating diverse evidence to provide a systemic understanding of community interventions and their links to public policy.

### 4.4. Limitations

This review presents several limitations that should be considered when interpreting the findings. First, a potential selection bias may be present, as study inclusion depended on the availability of full-text articles and publication in indexed journals, despite the use of a comprehensive search strategy. This may have led to the exclusion of relevant evidence, particularly from local contexts or community-based interventions not captured in international databases.

Second, the exclusion of grey literature (e.g., technical reports, institutional documents, theses, and program evaluations) may have limited the inclusion of practical implementation experiences, especially in low- and middle-income settings where such interventions are often not published in academic journals.

Third, the restriction to studies published in Spanish, English, and Portuguese introduces potential language bias, which may have excluded relevant research from other cultural and geographical contexts.

Fourth, an important limitation relates to the heterogeneity of the included studies. The inclusion of diverse methodological designs—quantitative, qualitative, mixed-methods, as well as secondary reviews—introduces variability in analytical approaches, populations, and outcome indicators, which limits direct comparability across studies and may affect the internal consistency of the synthesis.

Fifth, the inclusion of both primary studies and secondary reviews may introduce a risk of evidence overlap. Although analytical strategies were applied to distinguish sources and minimize duplication, its influence cannot be entirely ruled out.

Sixth, limited reporting on key variables such as sustainability, financing, implementation fidelity, and long-term outcomes constrains a comprehensive assessment of the impact of community-based interventions.

Finally, the nature of scoping reviews, while allowing for a broad mapping of evidence, is not designed to establish causal relationships or provide precise estimates of effectiveness. Accordingly, the findings should be interpreted as a synthesis of patterns and trends rather than definitive conclusions. Although the review focused on studies published between 1990 and 2024, a limited number of recent documents published in 2025 were included exclusively to provide updated policy context and were not part of the formal evidence synthesis.

Despite these limitations, the methodological diversity is consistent with the purpose of scoping reviews, which aim to capture the complexity and breadth of available evidence. To mitigate these challenges, a narrative and analytical synthesis approach was adopted, prioritizing the identification of common patterns, implementation mechanisms, and links with health systems and policy frameworks over direct comparisons of results.

### 4.5. Future Research

Strengthening applied research in community mental health is recommended, prioritizing longitudinal designs, impact evaluations, and cost-effectiveness analyses. Recent evidence [55] indicates that although effective programs based on cognitive behavioral therapy, resilience, mindfulness, or the promotion of healthy lifestyles exist, methodological rigor is often low, and only a minority incorporate sustained evaluations over time.

Studies such as InSHAPE [56,57] demonstrate initial improvements in lifestyles and mental well-being but also reveal a loss of impact following the end of funding [58], underscoring the need to incorporate sustainability from the design phase. In Latin America, an Ibero-American review [59] found that most public mental health policies are concentrated within the health sector, lacking multisectoral evaluation and interinstitutional monitoring mechanisms. Likewise, a PAHO report [60] highlights the urgency of “building back better” approaches, while noting a persistent evidence gap regarding their actual implementation.

### 4.6. Implications for Colombia

In the Colombian context, the findings support the need to formally integrate community-based mental health promotion into national health plans under intersectoral, participatory, and human rights-based approaches. Several studies propose relevant frameworks for this integration, including the proportionate universalism approach [46] and the focus on social, cultural, and structural determinants of mental health [47,49].

Intersectoral coordination also emerges as a critical factor, as recent studies describe disruptions between hospital-centered medical discourse and community dynamics, generating gaps in trust and power [59]. Although validated instruments exist to measure intersectoral collaboration, their use in Latin America remains limited [61]. Socio-occupational interventions, when reduced to isolated actions without follow-up, show low effectiveness and may increase the risk of revictimization [62].

## 5. Conclusions

In the Colombian context, the findings of this scoping review confirm the strategic value of community-based mental health promotion interventions as a complement to formal care systems. In particular, the results reinforce the need to systematically articulate these strategies with the National Mental Health Policy, the renewed Primary Health Care approach, and the existing regulatory frameworks for the care of psychoactive substance use [19,63].

This integration requires clear intersectoral governance arrangements among the health, education, employment, and social protection sectors, as well as the incorporation of structural, process, and outcome indicators to enable monitoring of the effectiveness and sustainability of interventions in the medium term [53].

Additionally, the results of this review highlight the importance of systematic processes for contextual adaptation of international evidence in community mental health promotion. The reviewed literature indicates that the uncritical transfer of programs may limit their effectiveness, whereas planned adaptation approaches—such as those proposed by the ADAPT and CHRODIS PLUS guidelines—increase the relevance, acceptability, and sustainability of interventions [64,65,66].

In this regard, participatory community-based work plays a key role in translating available evidence into territorial realities, respecting local beliefs, values, and resources, and promoting continuity of care across diverse community contexts [61].

Overall, this scoping review provides a comprehensive foundation to inform the planning, implementation, and evaluation of community-based mental health promotion strategies, both in Colombia and in other contexts facing similar challenges.

In this context, the conclusions should be understood as an overview of general trends in how community-based mental health strategies are implemented and integrated, rather than as definitive evidence of their comparative effectiveness. This underscores the need for future studies with more consistent designs and longitudinal approaches to better assess their impact.

## Figures and Tables

**Table 1:** General characteristics of the included studies (*n* = 76).

Characteristic	Result
Total number of included studies	76
Countries represented	16
Publication period	1990–2024
Type of review	Scoping review
Eligibility framework	Population–Concept–Context (PCC)
Main focus	Community-based mental health promotion strategies
Reported contexts	School-based, community, digital, workplace, cultural, others

Source: Authors’ own elaboration based on the scoping review conducted. Note: The methodological and conceptual heterogeneity identified across the studies is consistent with the exploratory and descriptive nature of the field of community mental health promotion.

**Table 2:** Summary of included studies by key characteristics, intervention types, and outcomes (*n* = 76).

Dimension	Findings
Geographic distribution	Studies were conducted across diverse regions, including Europe (Spain, UK, Portugal), Asia (India, Taiwan, Malaysia), North America (USA, Canada), Latin America (Brazil), Africa (Uganda), and international/multiregional contexts.
Type of intervention	Interventions included school-based programs (e.g., socio-emotional learning, resilience), community-based participatory interventions (e.g., peer support, arts-based programs), workplace initiatives, digital tools, physical activity programs, and culturally adapted strategies.
Target populations	The most frequent populations were children and adolescents, followed by adults, older adults, vulnerable populations (e.g., Indigenous youth, low-resource settings), and community members in general.
Study designs	A wide range of methodologies was identified, including controlled trials, quasi-experimental studies, quantitative pre–post designs, qualitative studies, and systematic/scoping reviews.
Key outcomes	Most studies reported improvements in well-being, resilience, psychosocial skills, and social support. Additional outcomes included reduced anxiety and depressive symptoms, improved knowledge and behaviors, and strengthened community cohesion.
Implementation components	Common components included community participation, facilitator training, peer support, cultural adaptation, and intersectoral collaboration.
Policy integration	Several studies highlighted links with public health frameworks, including primary health care approaches, social determinants of health, and community mental health models, although integration remained uneven.
Methodological quality	The majority of studies were classified as high or medium–high quality, with fewer studies rated as medium or low, reflecting variability in methodological rigor across designs.

Source: Authors’ own elaboration based on the scoping review conducted.

## Data Availability

No new data were created or analyzed in this study.

## Supplementary Materials

The following supporting information can be downloaded at https://www.mdpi.com/article/10.3390/healthcare14131931/s1: File S1: Researchers Results; File S2: Search Strategy and Reproducibility.
