# Supplementary material for: Community-Based Mental Health Promotion and Public Policy Integration: A Scoping Review (1990–2024)
Source: Healthcare (Basel). 2026 Jul 1;14(13):1931. doi: 10.3390/healthcare14131931 (PMC13362382; doi:10.3390/healthcare14131931)
Supplement: Supplementary file 1 [file healthcare-14-01931-s001.zip › Supplementary Material File S1 Researchers Result.pdf]

Supplementary Material File S1. Researchers Results

Researcher 1 Results

Author	Year	Country	Type of Intervention	Population	Results	Methodology	Quality
Abd Razak et al. (2024)	2024	Malaysia	Sanubari OHP Program	Young physicians	Improves self-efficacy and reduces anxiety among young physicians	Quasi-experimental with control group	Medium–High
Borgi et al. (2020)	2020	Portugal	Systematic review on community gardens	Non-institutionalized individuals	Improves social skills and well-being in rural settings	Quantitative pre–post	Medium–High
Calear et al. (2016)	2016	Australia	Sources of Strength Australia Project	Adolescent students	Strengthens networks and help-seeking behaviors	Controlled trial	High
Caretti Giangaspro et al. (2019)	2019	Spain	Childhood intervention	Children	Warns of risks of non-contextualized preventive interventions	Systematic review	High
Cejudo et al. (2020)	2020	Spain	*Aislados* Program	Adolescents	Video game improves well-being and socio-emotional learning	Quasi-experimental with control group	Medium–High
Chi et al. (2021)	2021	Taiwan	Multidisciplinary active aging intervention	Older adults	Active intervention improves mental health and social participation	Controlled trial	High
de Deuge et al. (2020)	2020	Australia	Community-based resilience mental health promotion program	Adults in rural communities	Community resilience facilitates program implementation	Applied qualitative study	Medium
Fairclough et al. (2024)	2024	United Kingdom	School-based motor competence intervention	School students	Improves motor competence and well-being	Quantitative and systematic reviews	Medium–High
Fanian et al. (2015)	2015	United States	Participatory program for Indigenous youth	Indigenous youth	Art strengthens resilience and social bonds	Applied qualitative study	Medium
Fenwick-Smith et al. (2018)	2018	Australia	School-based resilience promotion programs	School-age children	Increases child resilience and well-being	Systematic review	High
Gaiha et al. (2021)	2021	United Kingdom	Community mental health-focused interventions	Diverse population	Art reduces stigma and improves youth attitudes	Systematic review	High
Hardes (2018)	2018	United Kingdom	Physical exercise	Women	Exercise challenges stigma and promotes women's well-being	Applied qualitative study	Medium
Hartmann et al. (2018)	2018	Canada	Museum-based artistic intervention	Older adults	Hope-based strategy reduces psychological risk	Quantitative pre–post	Medium–High

Henteleff & Wall (2018)	2018	Canada	HANS KAI Project	Adults in local communities	Peer support improves health and community cohesion	Applied qualitative study	Medium
Hinrichsen et al. (2020)	2020	Denmark	ABC mental health promotion initiative	General population	Assesses implementation and acceptance of ABC initiative	Applied qualitative study	Medium
Jarman et al. (2016)	2016	Australia	Healthy@Work	Workers	Improves well-being and reduces work-related stress	Quantitative pre–post	Medium–High
Jessiman et al. (2022)	2022	United Kingdom	School culture and student mental health	Secondary school students	School culture influences student mental health	Applied qualitative study	Medium
Jiménez et al. (2016)	2016	Spain	Well-being program for older adults	Older adults	Positive psychology improves well-being	Quantitative and systematic reviews	Medium–High
Juliá-Sanchis et al. (2020)	2020	Spain	Community mental health care model	General population	Describes evolution of the community mental health model	Systematic review	High
Lampert et al. (2021)	2021	Portugal	Systematic review on community gardens	Non-institutionalized individuals	Community gardens improve physical and mental health	Systematic review	High
Las Hayas et al. (2019)	2019	Brazil	Modern postural yoga for mental health promotion	Adults	UPRIGHT increases resilience and well-being in students	Controlled trial	High
Mackenzie & Williams (2018)	2018	United Kingdom	Universal school-based interventions	School students	Improves emotional well-being	Systematic review	High
McAllister et al. (2018)	2018	Australia	Universal youth mental health promotion program	Regional youth	Increases resilience in regional youth	Quantitative pre–post	High
Mountain et al. (2017)	2017	United States	Workplace exercise program	Employees	Healthy lifestyles improve well-being in older adults	Controlled trial	High
Schwager et al. (2019)	2019	United States	Community behavioral interventions	Adults in local communities	School-based tool improves student mental health	Quantitative pre–post	Medium–High
Shankar et al. (2020)	2020	India	Youth-led school educational program	Female adolescents	Youth education improves knowledge and healthy practices	Quantitative pre–post	Medium–High

Researcher 2 Results

Author	Year	Country	Type of Intervention	Population	Results	Methodology	Quality
Díez et al.	2014	Spain	Community program to reduce isolation among older adults	Urban older adults	Improved perceived mental health and reduced isolation	Quasi-experimental with control group	High
Moeller-Saxone et al.	2015	Asia-Pacific	Systematic review on promotion in LMICs	General	Identifies gaps, barriers, and effective community promotion strategies	Systematic review	High
Welsh et al.	2015	International	Scoping review on equity in child mental health	Children and adolescents	Proposes an equity and proportional universalism framework	Scoping review	High
Barry et al.	2007, 2009, 2013	Ireland, UK	School-based mental health promotion programs	Schoolchildren	Improves socioemotional competencies, resilience, and school climate	Quantitative and systematic reviews	High
Jané-Llopis et al.	2005, 2007	Europe	Comparative review of youth promotion and prevention	Youth	Differentiates promotion and prevention; intersectoral models	Systematic review	High
Hosman et al.	2005	Netherlands	Universal prevention with promotional components	Schoolchildren	Reduces anxiety and depressive symptoms; improves psychosocial skills	Controlled trial	Medium–High
Huang et al.	2014	Uganda	School-based intervention in low-resource settings	Schoolchildren	Teacher training improves behavior management and emotional promotion	Study protocol, experimental design	High
Shelton et al.	2006	USA	Community expressive arts program for vulnerable youth	African American youth	Improves resilience, self-control, and protective factors	Quantitative pre–post	High
Feather et al.	1993	Canada	Community development model in social health	Rural communities	Identifies structural barriers and local strategies	Applied qualitative study	Medium
