# Supplementary material for: Community-Based Mental Health Promotion and Public Policy Integration: A Scoping Review (1990–2024)
Source: Healthcare (Basel). 2026 Jul 1;14(13):1931. doi: 10.3390/healthcare14131931 (PMC13362382; doi:10.3390/healthcare14131931)
Supplement: Supplementary file 1 [file healthcare-14-01931-s001.zip › Supplementary Material File S2 Search Strategy and Reproducibility.pdf]

Supplementary Material File S2. Search Strategy and Reproducibility

References were searched for studies published between January 1, 1990, and December 31, 2024. The search was conducted in April 2025, between April 20 and April 25. A systematic search was conducted across the following databases: PubMed, Scopus, SciELO, the Virtual Health Library (Latin America), and Cochrane. Search terms were defined using MeSH and DeCS descriptors, as well as relevant synonyms, combined through Boolean operators (AND, OR) to ensure comprehensiveness. The groups of terms were organized according to the variables of interest:

The search terms were organized into the following thematic groups, combining DeCS and MeSH descriptors:

• Explanatory variables in mental health promotion
  - DeCS: Factores de Riesgo; Promoción de la Salud; Salud Mental
  - MeSH: Risk Factors; Health Promotion; Mental Health

• Public policies in mental health
  - DeCS: Política de Salud; Salud Mental
  - MeSH: Health Policy; Mental Health

• Theoretical models of mental health
  - DeCS: Modelos Teóricos; Salud Mental
  - MeSH: Theoretical Models; Mental Health

• Community-based mental health interventions
  - DeCS: Intervención Comunitaria; Salud Mental
  - MeSH: Community Intervention; Mental Health

• Social determinants of mental health
  - DeCS: Determinantes Sociales de la Salud; Salud Mental
  - MeSH: Social Determinants of Health; Mental Health

• National and international approaches to mental health
  - DeCS: Salud Mental; Política de Salud
  - MeSH: Mental Health; Health Policy

• Mental health promotion in vulnerable populations
  - DeCS: Promoción de la Salud; Grupos Vulnerables; Salud Mental
  - MeSH: Health Promotion; Vulnerable Populations; Mental Health

• Mental health strategies within public policy frameworks
  - DeCS: Estrategias; Política de Salud; Salud Mental
  - MeSH: Strategies; Health Policy; Mental Health

For the Cochrane database, search equations were constructed using both explanatory and general variables, employing MeSH descriptors combined with Boolean operators. The search strategy was as follows:

(("Risk Factors"[MeSH Terms]) AND ("Health Promotion"[MeSH Terms]) AND ("Health Policy"[MeSH Terms]) AND ("Mental Health"[MeSH Terms])) OR (("Theoretical Models"[MeSH Terms]) AND ("Mental Health"[MeSH Terms]) AND ("Community Intervention"[MeSH Terms])) OR (("Social Determinants of Health"[MeSH Terms]) AND ("Mental Health"[MeSH Terms]) AND ("Health Policy"[MeSH Terms])) OR (("Mental Health"[MeSH Terms]) AND ("Health Policy"[MeSH Terms] OR "Health Promotion"[MeSH Terms])) OR (("Community Intervention"[MeSH Terms]) AND ("Mental Health"[MeSH Terms]) AND ("Health Policy"[MeSH Terms])) OR (("Mental Health"[MeSH Terms]) AND ("Vulnerable Populations"[MeSH Terms]) AND ("Health Promotion"[MeSH Terms]))

The Scopus search was performed using the following equations, applying English descriptors combined with Boolean operators:

1. ("Risk Factors" AND "Health Promotion" AND "Health Policy" AND "Mental Health")
2. ("Theoretical Models" AND "Mental Health" AND "Community Intervention")
3. ("Vulnerable Populations" AND "Health Promotion" AND "Mental Health")
4. ("Strategies" AND "Health Promotion" AND "Mental Health")
5. ("Social Determinants of Health" AND "Mental Health" AND "Health Promotion" AND "Disease Prevention")
6. ("Community Intervention" AND "Mental Health" AND "Health Policy")

The PubMed search was conducted through individual queries, organized according to explanatory variables, using MeSH and DeCS descriptors combined with Boolean operators. The strategies were as follows:

1. (“Factores de Riesgo” AND “Promoción de la Salud” AND “Política de Salud” AND “Salud Mental”)
2. (“Modelos Teóricos” AND “Salud Mental” AND “Intervención Comunitaria”)
3. (“Determinantes Sociales de la Salud” AND “Salud Mental” AND “Política de Salud”)
4. (“Salud Mental” AND (“Política de Salud” OR “Promoción de la Salud”))
5. (“Intervención Comunitaria” AND “Salud Mental” AND “Política de Salud”)
6. (“Salud Mental” AND “Grupos Vulnerables” AND “Promoción de la Salud”)

The SciELO search strategy was conducted using the following six equations:

1. (“Factores de Riesgo” AND “Promoción de la Salud” AND “Política de Salud” AND “Salud Mental”)
2. (“Modelos Teóricos” AND “Salud Mental” AND “Intervención Comunitaria”)
3. (“Determinantes Sociales de la Salud” AND “Salud Mental” AND “Política de Salud”)
4. (“Salud Mental” AND (“Política de Salud” OR “Promoción de la Salud”))
5. (“Intervención Comunitaria” AND “Salud Mental” AND “Política de Salud”)

6. (“Salud Mental” AND “Grupos Vulnerables” AND “Promoción de la Salud”)

All combinations were defined to ensure comprehensive identification of studies related to mental health promotion in community settings.

Search Strategy and Reference Management

Reference management was conducted using Mendeley software, ensuring systematic organization, accurate citation, and removal of duplicates. This process was carried out between April 20 and April 25, 2025.

The previously defined MeSH and DeCS terms were applied, yielding a total of 3,799 records identified across the different databases. These records were organized into folders within the reference manager.

The distribution of records, subsequently assigned to the researchers, was as follows: Scopus (n = 684), Cochrane (n = 43), Virtual Health Library (VHL) (n = 2,966), PubMed (n = 10), SciELO (n = 32), and other sources (n = 64).
